# The influence of smoking in minimally invasive spinal fusion surgery

**DOI:** 10.1515/med-2021-0223

**Published:** 2021-01-27

**Authors:** Wolfgang Senker, Harald Stefanits, Matthias Gmeiner, Wolfgang Trutschnig, Christian Radl, Andreas Gruber

**Affiliations:** Department of Neurosurgery, Kepler University Hospital, Neuromed Campus, Linz, Austria; Department of Mathematics, University of Salzburg, Salzburg, Austria

**Keywords:** smoking, minimally invasive fusion techniques, lumbar spine, complication rate

## Abstract

**Background:**

The impact of smoking on spinal surgery has been studied extensively, but few investigations have focused on minimally invasive surgery (MIS) of the spine and the difference between complication rates in smokers and non-smokers. We evaluated whether a history of at least one pack-year preoperatively could be used to predict adverse peri- and postoperative outcomes in patients undergoing minimally invasive fusion procedures of the lumbar spine. In a prospective study, we assessed the clinical effectiveness of MIS in an unselected population of 187 patients.

**Methods:**

We evaluated perioperative and postoperative complication rates in MIS fusion techniques of the lumbar spine in smoking and non-smoking patients. MIS fusion was performed using interbody fusion procedures and/or posterolateral fusion alone.

**Results:**

Smokers were significantly younger than non-smokers. We did not encounter infection at the site of surgery or severe wound healing disorder in smokers. We registered no difference between the smoking and non-smoking groups with regard to peri- or postoperative complication rate, blood loss, or length of stay in hospital. We found a significant influence of smoking (*p* = 0.049) on the overall perioperative complication rate.

**Conclusion:**

MIS fusion techniques seem to be a suitable tool for treating degenerative spinal disorders in smokers.

## 1 Introduction

Smoking continues to contribute considerably to health problems across Europe. According to Eurostat, 19.2% of Europeans smoke on a daily basis, whereas 4.7% are occasional smokers. In the European Union (EU), one in four people (24.9%) aged 15 years or older smokes with a higher percentage of male smokers (28.7%) than female smokers (19.5%). Consequently, spine surgeons have to treat a sizable group of smoking patients. Moreover, smoking has been shown to have a harmful effect on bone healing. The literature shows that the rate of nonunion or pseudarthrosis after spinal fusion is higher in smokers than in non-smokers [1,2,3,4,5]. However, the distinct pathophysiologic mechanisms that lead to these differences are still unclear. The most commonly accepted theories hypothesize that a decrease in systemic bone mineral density, osteoblastic cellular metabolism, local blood flow, and angiogenesis may cause these differences [6]. Smokers are also significantly more likely to report less favorable clinical outcomes after spinal surgery. Furthermore, the literature shows that smokers suffer from postoperative infections more frequently than non-smokers do [3]. This study compares the perioperative complication rate, blood loss, and length of hospital stay of smoking and non-smoking patients who are undergoing minimally invasive fusion procedures of the lumbar spine. Data treating these specific topics, especially in minimally invasive surgery (MIS), are scarce.

## 2 Materials and methods

This study was approved by the ethics committee. We recruited 187 patients for this prospective investigation, of which 115 were female and 72 male. Written informed consent was obtained from all patients, and the study was registered at ClinicalTrials.gov (NCT01259960). We categorized patients as smokers (history of at least one pack-year) and non-smokers and further subdivided them according to age group. We split the data into two age groups (≤64 and ≥65).

### 2.1 Surgical technique

Revisions of the disc space and laminotomy for spinal stenosis were performed using the Quadrant Tubular Retractor System (Medtronic Inc., Memphis, TN, USA). After identifying the appropriate facet joint using fluoroscopy, an incision was made 1.5 cm off the midline. A tube was inserted subcutaneously and muscle tissue was sequentially dilated by creating a corridor to the facet joint in a fashion similar to that described by Foley and Smith [7]. Next, a tubular retractor was inserted. The facet joint and the yellow ligament were exposed. We used the percutaneous fusion system Sextant II or Longitude (both Medtronic Inc.) for posterolateral fusion. In 360° fusion cases, we performed a transforaminal lumbar interbody fusion (TLIF) procedure [8]. In spinal stenosis cases, the retractor was directed to the contralateral side of the spinal canal to perform a laminotomy (146 patients) [9].

### 2.2 Statistics

Statistical analyses were performed using the R package (npmv). We used the nonpartest [10] for testing the null hypothesis that the underlying distributions in the groups under investigation coincided. Whenever three groups were encountered, we used the standard three-sample test for equality of proportions. A linear dependence of variables was determined by Pearson’s correlation, whereas concordance was demonstrated by Spearman’s rank correlation. Statistical significance was assumed by a *p*-value of <0.05.

## 3 Results

**Figure 1:**
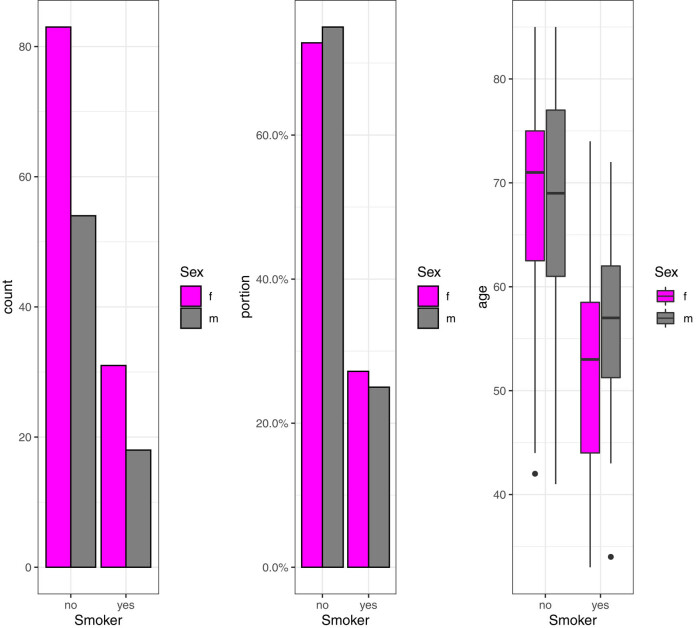
Number of female and male smokers (left and middle panels) and age distribution of smokers and non-smokers (right panel).

The sample of 187 patients contained 18 male (25%) and 31 female (27%) smokers (for one patient, no information was available). The mean age of the total cohort was 64.27 years (range: 33–85 years), that of the smoker group was 53.27 years (49–74 years), and that of the non-smoker group was 68.10 years (41–85 years). The age distributions of smokers and non-smokers differ significantly (Figure 1). Going forward, we will refer to this fact as “smoking-age-bias.” Testing for equal age distribution in the smoking and the non-smoking groups using nonpartest [10] yields a *p*-value of 0 for the full sample, as well as for the male and the female subsamples. Consequently, any variable of interest that positively correlates with the “age” variable is likely to have lower correlation values in the smoker group than in the non-smoker group. Furthermore, we defined diabetes (DM, *n* = 34), coronary heart disease (CHD, *n* = 20), any other cardiac disease including atrial fibrillation (*n* = 9), and peripheral vascular disease (PAD, *n* = 7) as secondary diseases and looked at a possible statistical impact. Fifty-eight patients suffered from at least one secondary disease.

### 3.1 Surgery-related complications

**Figure 2:**
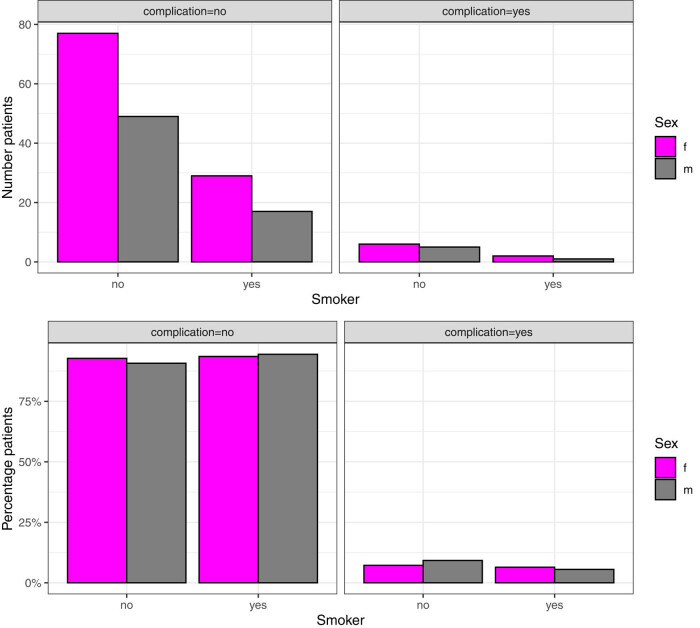
Number and percentage of smokers with and without perioperative complications.

Since the following complications are directly related to surgery, we defined them as surgery-related complications. The study registered 14 patients who experienced surgery-related complications (6.95%): 3 in the smoker group (6.12%) and 11 in the non-smoker group (8.03%). Of these, 12 had to undergo revision (4.87%). These complications included: one impression fracture of an L4 endplate in a 360° fusion of L3–L5; one iatrogenic fracture of an S1 pedicle; one excoriation due to the removal of surgical drapes; one extraforaminal hematoma with a persistent neurological deficit; three epidural hematomas (one patient was revised on day 2, one on day 4, and one on day 5); one patient with a screw malposition, who had to undergo revision; one patient with activated arthritis of the shoulder due to inappropriate positioning on the operating table; and one with a loosened screw (due to osteoporosis), which occurred 2 months postoperatively and needed revision. Furthermore, one patient had to undergo revision because of a weakness of dorsiflexion of the foot due to a bone fragment in the spinal canal. Another patient had to undergo revision because of a pedicle fracture that caused a space-occupying lesion in the spinal canal and one because of a dislocation of a TLIF. There were 3 patients (1 male and 2 females) in the smoker group and 11 patients (5 males and 6 females) in the non-smoker group, who suffered from surgery-related complications (Figure 2). This is consistent with the previously reported results, which indicate that the surgery-related complication rate was slightly lower in the smoker group. Nevertheless, we could not observe a statistically significant difference (*p* = 0.9).

### 3.2 Smoking versus perioperative complications

We combined wound healing disorders (WHDs), hematomas, wound dehiscence, tension cavities, and incidental durotomies into perioperative complications. We encountered one patient (0.534%) with WHDs. Two patients (1.07%) developed a wound dehiscence, one of which needed a secondary suture, and nine patients had hematomas (4.81%). No patient in this group had to undergo a revision in the operating room. In 22 patients (11.64%), we recorded liquor leakages. We could not see a statistically significant difference between the smoker and the non-smoker groups (*p* = 0.46) for all four of these perioperative complications.

### 3.3 Smoking versus overall perioperative complication percentages (i.e., cerebrospinal fluid leakage, surgery-related complications, WHD, dehiscence, wound necrosis, hematoma, or tension cavity)

We coded this group as a binary variable. Adjusting for age to avoid the smoking-age-bias, considering only the group of patients not older than 64, and testing for equal overall perioperative complication rates in the smoker and the non-smoker groups (6 out of 45 smokers and 11 out of 40 non-smokers had at least one perioperative complication), we found no statistically significant difference (*p* = 0.17). We recognized DM, CHD, PAD, and cardiac disease as secondary diseases. Considering that secondary diseases might potentially lead to perioperative complications and considering the smoke-age-bias, we calculated two logistic regression models: (i) one with overall perioperative complication (yes = 1 and no = 0) as binary output and overall secondary disease (yes/no) as well as age and smoking (yes/no) as explanatory variables. Neither smoking (*p* = 0.080) nor overall secondary disease (*p* = 0.529) showed a statistically significant influence on the perioperative complication rate. (ii) Avoiding aggregation of secondary diseases, age, smoking, and each of the four aforementioned secondary diseases as separate explanatory variables in a multivariate logistic regression with perioperative complication (yes = 1 and no = 0) as binary output, we found a statistically significant influence of smoking (*p* = 0.049). However, we were not able to find any statistically significant impact of DM (*p* = 0.527), CHD (*p* = 0.349), PAD (*p* = 0.297), or cardiac diseases (*p* = 0.592) on the complication rate.

### 3.4 Smoking versus postoperative complications

We grouped any adverse event in the postoperative period under “postoperative complications.” In total, 18 possible postoperative complications were recorded for 186 patients; data were missing for 1 patient. Eighteen patients (9.68%) had a fever, whereas 45 (24.19%) had subfebrile temperatures. Seven patients (3.76%) had a urinary tract infection and two (1.08%) had pneumonia. One patient (0.54%) sustained fatal pulmonary emboli. Thirty patients (16.13%) had a neurological deficit, which included any form of self-reported transient sensation. Four patients had atrial fibrillation postoperatively (2.15%), two (1.08%) had cardiac ischemia, and one (0.54%) had a transient ischemic attack. Two patients had a myocardial infarction (1.08%), six had anemia (3.23%), and one patient each had enteritis, urinary retention, reflux esophagitis, (pre-)ileus, and an attack of gout (0.54%) (Table 1). Overall, postoperative complication percentages increase with age, implying that the smoking-age-bias might lead to lower percentages in the smoker group. The fact that overall postoperative disease percentages increase with age implies that the smoking-age-bias might lead to lower percentages in the smoker group. Only in pneumonia and subfebrile cases did the smoker group show (slightly) higher percentages. Nevertheless, we could not find any statistically significant differences between the smoker and the non-smoker groups for each postoperative complication. Testing for equal postoperative complication percentages in the smoker and the non-smoker groups, we could not find any statistical differences (*p* = 0.57).

**Table 1:** Number and percentage of postoperative complications in the smoker and the non-smoker groups

Complication	Smoker	No of patients	Percentage
Anemia	No	6	0.0438
Anemia	Yes	0	0
Atrial fibrillation	No	3	0.0219
Atrial fibrillation	Yes	1	0.0204
Attack of gout	No	1	0.0073
Attack of gout	Yes	0	0
Cardiac ischemia	No	2	0.0146
Cardiac ischemia	Yes	0	0
Enteritis	No	1	0.0073
Enteritis	Yes	0	0
Ileus/preileus	No	1	0.0073
Ileus/preileus	Yes	0	0
Meningismus	No	0	0
Meningismus	Yes	0	0
Myocardial infarction	No	2	0.0146
Myocardial infarction	Yes	0	0
Neurological deficit	No	24	0.1752
Neurological deficit	Yes	6	0.1224
Pneumonia	No	1	0.0073
Pneumonia	Yes	1	0.0204
Pulmonary emboli	No	1	0.0073
Pulmonary emboli	Yes	0	0
Reflux oesophagitis	No	1	0.0073
Reflux oesophagitis	Yes	0	0
Respiratory tract infection	No	0	0
Respiratory tract infection	Yes	0	0
Subfebrile	No	32	0.2336
Subfebrile	Yes	13	0.2653
Temperature	No	15	0.1095
Temperature	Yes	3	0.0612
Transient ischemic attack	No	1	0.0073
Transient ischemic attack	Yes	0	0
Urinary retention	No	1	0.0073
Urinary retention	Yes	0	0
Urinary tract infection	No	6	0.0438
Urinary tract infection	Yes	1	0.0204

**Figure 3:**
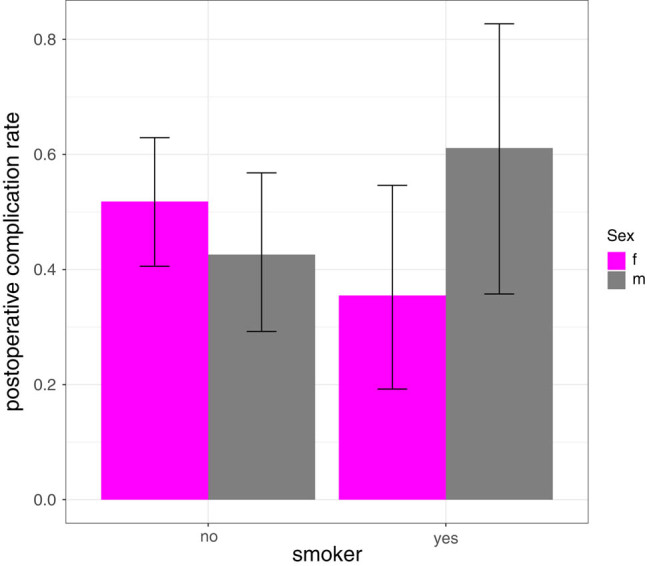
The postoperative complication rate in female and male smokers.

The probability of occurrence of at least one postoperative complication was not different between the smoker and the non-smoker groups (44.90 vs 48.18%, *p* = 0.7). Interestingly, we saw in the (smaller) male group a higher complication rate in the smoker group (61.11 vs 42.59%). In contrast, in the (larger) female group the complication was lower in the smoker group (35.48 vs 51.81%) (Figure 3). Although there is a stronger smoking-age-bias in the female group (approximately 3 years), this fact only partially explains the substantial difference. Adjusting for age to avoid the smoking-age-bias, considering only the group of patients not older than 64, and testing for equal overall complication rates in the smoker and the non-smoker groups (19 out of 45 smokers and 15 out of 40 non-smokers had at least one postoperative complication), we found no statistically significant difference (*p* = 0.82). Considering that secondary diseases might potentially lead to complications and considering the smoke-age-bias, we calculated two logistic regression models: (i) one with overall postoperative complication (yes = 1 and no = 0) as binary output and overall secondary disease (yes/no) as well as age and smoking (yes/no) as explanatory variables. Neither smoking (*p* = 0.642) nor overall secondary disease (*p* = 0.866) showed a statistically significant influence on the postoperative complication rate. (ii) Avoiding aggregation of secondary diseases, age, smoking, and each of the four aforementioned secondary diseases as separate explanatory variables in a multivariate logistic regression with postoperative complication (yes = 1 and no = 0) as binary output, we did not find any statistically significant influence of smoking (*p* = 0.114), DM (*p* = 0.858), CHC (*p* = 0.997), PAD (*p* = 0.074), or cardiac disease (*p* = 0.344).

### 3.5 Smoking versus blood loss

We defined blood loss as the sum of blood loss during surgery, the monitoring phase, and the postoperative drainage phase. Mean blood loss during the surgery and monitoring phase was 93.54 (0–1,050 ml) in the entire sample, 79.38 ml (0–1,050 ml) in the smoking group, and 98.54 ml (0–1,000 ml) in the non-smoking group. Blood loss per drainage was 143.13 ml (0–790 ml) in the collective and 116.77 ml (0–410 ml) and 152.50 ml (0–790 ml) in the subgroups. Total blood loss was 237.18 ml (0–1,600 ml), 196.14 ml (0–1,150 ml), and 251.77 ml (0–1,600 ml). Age and blood loss (perioperative and monitoring) are weakly correlated (*ρ* ≐ 0.13, *ρ*s ≐ 0.15) as are age and amount of drainage (*ρ* ≐ 0.18 and *ρ*s ≐ 0.13). As a direct consequence of this fact, and given the smoking-age-bias, the smoker group shows a tendency toward less blood loss/drainage than the non-smoker group. Since testing for equal blood loss/drainage distributions and ignoring the smoking-age-bias might lead to wrong conclusions, we only focused on the descriptive statistics.

### 3.6 Smoking versus discharge day

**Figure 4:**
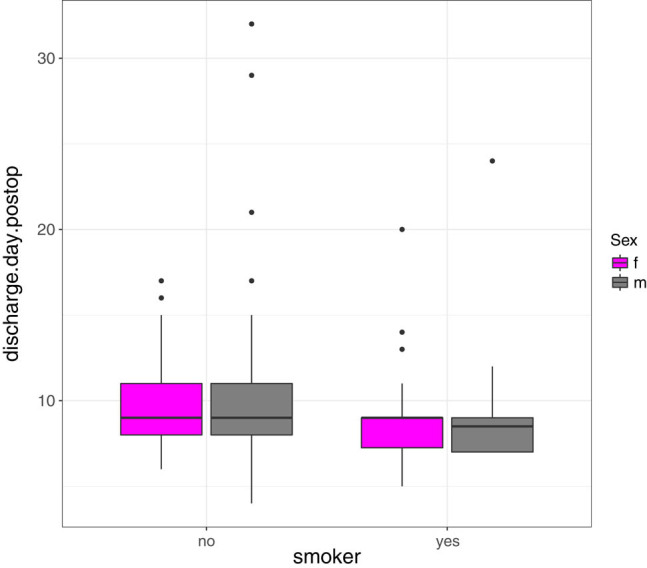
Comparison of the discharge day in female and male smokers as well as non-smokers.

Data for two patients were missing. The average length of stay (LOS) in hospital was 9.59 days (4–32 days), 9.02 days (5–24 days) in the smoker group, and 9.79 days (4–32 days) in the non-smoking group. For age and discharge day, Pearson correlation *ρ* and Spearman rank correlation *ρ*s are given as *ρ* ≐ 0.1181 and *ρ*s ≐ 0.1994. Testing for the null hypothesis of zero Pearson (Spearman) correlation yields a *p*-value of 0.1101 (0.006656). As a result of the age bias, we saw a tendency that the average LOS in the smoking patient cohort is shorter than that of the average non-smokers (Figure 4). Again, as ignoring the smoking-age-bias might lead to wrong conclusions, we focused on the descriptive statistics.

## 4 Discussion

In this study, we investigated the perioperative complication rate in smokers undergoing minimally invasive fusion surgery. MIS techniques minimize soft tissue damage, reduce blood loss, show less postoperative pain, and a shorter hospital stay [11,12,13,14,15]. The impact of smoking on the general state of health is well known. Vogt et al. investigated the association between the smoking status of spinal surgery patients, the duration and severity of symptoms, and their self-reported health status. They found that smokers reported more severe symptoms than non-smokers [16]. Furthermore, those who were non-smokers reported better health status postoperatively than those who smoked. Smoking increases the risk of pseudarthrosis in both lumbar and cervical fusion procedures [1,2,3,4,5]. In this study, we focused on the perioperative effects of smoking tobacco in MIS fusion procedures of the spine. Turan et al. evaluated more than 5,00,000 patients to determine the effect of smoking on 30-day perioperative outcomes in noncardiac surgical patients [17]. In their study, current smokers were 1.38 times more likely to die than patients who had never smoked. They also had significantly greater odds of suffering from pneumonia, requiring unplanned intubation or needing mechanical ventilation. Current smokers were substantially more likely to experience a cardiac arrest, myocardial infarction, and stroke. Seicean et al. saw similar effects of smoking on the perioperative outcome of patients undergoing elective surgery [18]. They selected 14,500 patients who needed elective spinal surgery from the American College of Surgeons National Surgical Quality Improvement database and divided them into current, prior, and never smokers. Compared with both prior and never smokers, current smokers had fewer comorbidities, abnormal laboratory values, and intra/postoperative transfusions. Previous smokers were older than current and never smokers, had more comorbidities and abnormal lab values, and were less likely to be operated on by the attending surgeon alone. Prior smokers were also found to have a significantly greater number of major complications than never smokers, with 7 and 5.4% being affected, respectively. Current smokers with more than 60 pack-years were more likely than never smokers to die within 30 days of surgery. However, smoking itself was not found to be associated with poorer operative or 30-day outcomes in nearly all patients undergoing elective spinal surgery. De la Garza Ramos et al. investigated 1,368 patients who underwent surgery for adult spinal deformity and saw no significant difference between smokers and non-smokers when it came to the development of complications, either in major complication rates or increased odds of developing any complication or major complication [19]. In our group, we could not detect any significant difference between smokers and non-smokers when it came to surgery-related complications in our cohort. We could not identify any statistically significant differences between peri- or postoperative complications and smoking. In the overall perioperative complications group, we found a weak, statistically significant smoking influence (*p* = 0.049). Possible relevant secondary diseases such as DM, CHD, PAD, or cardiac diseases did not influence the results.

Due to the significantly lower average age of smokers in the sample and the fact that age has a weak positive correlation with peri- and postoperative complication rates, the corresponding complication rates were slightly lower in the smoker group. There was only a marginally greater number of patients suffering from subfebrile temperatures or pneumonia in the smoker group. Interestingly, we saw a higher rate of postoperative complications in the male smoker group (61.11 vs 42.59%), whereas in the larger female group, the rate of postoperative complications was lower in the smoker group (35.48 vs 51.81%). Tobacco smoking increases the risk of wound complications and infection by reducing tissue oxygenation and blood flow. Smoking also decreases the effectiveness of inflammatory cell function and oxidative bactericidal mechanisms. Furthermore, reparative cell functions are inhibited [20]. Nevertheless, the literature provides diverging positions concerning smoking and wound infection. Turan et al. found that current smokers had significantly higher odds of having superficial and deep incisional infections, sepsis, organ space infections, and septic shock [17]. Veeravagu et al., who studied 24,774 patients, reported that smokers had a statistically significant higher rate of infection than non-smokers (OR: 1.19, 95% CI: 1.02–1.37) [21]. On the other hand, Cizik et al., who worked with 1,532 patients, found that smoking was not a significantly contributing factor in surgical site infections (SSIs) [22]. Lee et al. developed a predictive model for the occurrence of SSI after spinal surgery [23]. Interestingly, a history of congenital heart failure was the greatest medical risk factor for SSI in multivariate analysis. The odds of SSI in these patients were 3.07 times higher than they were for those without congenital heart failure when adjusted for surgical invasiveness and diabetes. According to their analysis, a 65-year-old man with a history of rheumatoid arthritis and diabetes, undergoing an L4–L5 laminectomy and transforaminal interbody fusion, has an 11.84% chance of SSI requiring an operative debridement. Smoking was considered as a predictor variable but was not defined as a criterion for infections. Ee et al. investigated the medical records of 2,299 patients after TLIF procedures, laminectomies, or discectomies comparing MIS with open spinal surgery [24]. Patients undergoing open spinal surgery were 5.77 times more likely to suffer from an SSI than MIS approaches. Furthermore, diabetes, the number of levels operated on, and body mass index were predictive of an increased SSI risk. Smoking was not a risk factor for suffering from an SSI. We observed no severe WHDs in our patients. We noted one WHD (0.534%) due to dry necrosis of the wound margin, one case of wound dehiscence that needed a secondary suture, and four superficial WHDs in the form of tension cavities (2.139%). We observed no significant difference between the smoking and non-smoking groups, but we did observe slightly better results in the smoking group. Smoking is associated with longer hospital stays. In their survey of 160 patients who underwent anterior cervical corpectomy, Lau et al. found that current smokers were subject to higher complication rates (*p* < 0.001) and longer lengths of stay (*p* < 0.001) [1]. Seicean et al. divided their cohort of 14,500 adults into current, prior, and never smokers [18]. Mean LOS was 2 days for all three groups. Current smokers had a shorter interquartile range of 1–3 days, compared with 1–4 days for both prior and never smokers. Interestingly, former smokers were most likely to have prolonged LOS (30.2%), whereas current smokers were the least likely (22.2%). Patients smoking more than 60 pack-years had a higher likelihood of a prolonged LOS than never smokers. In our study group, the mean LOS was around 9 days in both the smoking and the non-smoking groups. We saw that the average smoker in our patient cohort had spent less time in hospital than the average non-smoker. Very little research has been carried out in a possible association between smoking and intraoperative blood loss and perioperative transfusion use in patients undergoing spinal surgery. McCunniff et al. investigated 581 lumbar decompression cases with or without fusion [25]. They found that smokers had an increased estimated blood loss compared with non-smokers (mean 328 ml more for each pack smoked per day; 95% CI: 249–407 ml; *p* < 0.001). They also found that smokers had a greater perioperative transfusion rate than non-smokers. In our cohort, the average smoker suffered less blood loss/drainage than the average non-smoker. To our knowledge, this is one of the largest single-center studies investigating MIS procedures. A limitation of this study might be the close relationship of young age and smokers. Nevertheless, because of this very relationship, it would be even more remarkable if more adverse events were observed in the smoking group. This aspect in particular was not observed among our cohort.

## 5 Conclusion

The negative impact of smoking on health is undeniable. In the EU, one person out of four is a smoker (Eurostat). Consequently, spine surgeons must treat a significant population of patients who smoke. In our cohort of 187 patients undergoing surgery in a single center, smokers did not show an elevated risk of perioperative complications in MIS fusion. The use of MIS fusion techniques does itself provide a low comorbidity, too [15]. We do, of course, recommend postoperative smoking cessation, since this helps to reverse the impact of cigarette smoking on outcomes following spinal fusion, particularly as regard to a lower nonunion rate or a higher return to work rate [4].
